# Exploring the potential of Carica Papaya Leaf Extract: a perspective on its effectiveness in ameliorating thrombocytopenia in dengue patients

**DOI:** 10.1080/20008686.2025.2456055

**Published:** 2025-01-23

**Authors:** Maliha Khalid, Muhammad Saad Khan, Erum Siddiqui, Aminath Waafira

**Affiliations:** Department of Medicine, Jinnah Sindh Medical University, Karachi, Pakistan; School of Medicine, The Maldives National University, Malé, Maldives

**Keywords:** Thrombocytopenia, dengue, Carica Papaya Leaf Extract

## Abstract

Background: Dengue fever (DF) is endemic in Pakistan, posing health risks. Recent flooding in 2022 and strong monsoon rains in 2024 have increased the possibility of an epidemic. It is an infectious disease having potentially severe outcomes including thrombocytopenia.

Discussion: Carica Papaya Leaf Extract (CPLE) has emerged as an off-label treatment option, showing promising results in increasing platelet counts and reducing hospital stays. However, a critical assessment of existing research reveals methodological flaws, hindering specific recommendations.

Conclusion: This perspective advocates for comprehensive research to evaluate the risks and benefits of CPLE as a potential remedy for thrombocytopenia associated with dengue fever. A robust investigation will inform clinical practice and guide healthcare decisions, contributing to improved patient outcomes in dengue-endemic areas.

## Introduction

According to the World Health Organization (WHO), Dengue is endemic in Pakistan, and the country reported the last significant outbreak was in 2019, with 53,498 cases and 95 deaths occurring between September and December 2019. A total of 48,906 cases, including 183 deaths that works out to a Case Fatality Rate (CFR) of 0.4%, have been reported in four provinces: Punjab, Khyber Pakhtunkhwa, Sindh, Balochistan and the federally administered Islamabad Capital Territory in Pakistan. Additionally, reports were from Azad Jammu and Kashmir autonomous territories. A total of 25,932 confirmed Dengue cases, including 62 deaths, yielding a CFR of 0.25%, were reported in Pakistan from 1 January to 27 September 2022. However, over 74% of these were reported in September. Industrial facilities in affected countries pointed to an earlier beginning of the surge around mid-June 2022, roughly coinciding with unprecedented flooding. The historic flooding has led to a rapid increase in cases this year. The WHO country office was assisting the government of Pakistan by funding to carry out response activities, including strengthening molecular laboratory and hospital-based disease surveillance at provincial endemic provinces and national level, using existing platforms for Integrated Disease Surveillance (IDS), field epidemiology as well Dengue surveillance cells. Over the past few decades, there has been a dramatic increase in Dengue cases worldwide. Not all cases of Dengue are reported because a good percentage of them are asymptomatic, mild and self-treated. Dengue is transmitted in over 128 countries, putting nearly half the world’s population (almost 4 billion people) at risk. It is now endemic in more than 100 countries in the WHO Regions of Africa, the Americas, the Eastern Mediterranean, Southeast Asia and the Western Pacific. The Americas, Southeast Asia and Western Pacific regions are the most seriously affected, with Asia representing around 70% of the global disease burden.

As per the WHO, Dengue is a viral infection transmitted to humans by Aedes aegypti. The chief vectors for its transmission are mosquito glands, mainly Aedes aegypti and, secondarily, A. albopictus [1]. Dengue Fever (DF): Virus- Dengue fever is caused by a virus that infects the mosquito and reproduces there. The four Dengue virus (denV) serotypes are named DEN 1–4. One serotype infection confers long-term homotypic but not heterologous immunity, able to occur at most four times. Secondary infection leads them to increase the risk of severe Dengue during hospitalization. Dengue infections usually manifest clinically as mild fever that spontaneously recovers.

Thrombocytopenia, the most commonly recognized Transfusion-related Acute Lung Injury (TRALI) complication, is mainly due to direct antibiotic binding of platelets, bone marrow suppression and ineffective maturation of megakaryocytes with rapid peripheral clearance. The clinical spectrum of Dengue infection varies from an uncomplicated febrile illness to a severe disease with organ dysfunction. In the severe forms, plasma leakage resulting in shock and organ failure occurs; bleeding can be life-threatening. Mortality in Dengue is generally the result of shock, refractory multi-organ dysfunction or uncontrolled bleeding. The mortality during plasma leakage is mainly due to the development of pulmonary edema in the recovery phase – a fact that could well be aggravated if fluid therapy were too liberal [1] The proximity of mosquito vector breeding sites to human habitation is a significant risk factor for Dengue virus infection. Dengue infection has no specific treatment, but early detection and access to proper medical care lowers fatality rates [2]. In addition, the prevention and control of Dengue rely entirely on sound, sustained vector control measures. Despite decades of intensive investigation, relatively few established therapeutic targets for Dengue exists. Corticosteroids [3] and immunoglobulins [4] in immunosuppression have not been shown to benefit. While several antiviral agents and other host immune modulators are in various phases of clinical studies, they will not be available clinically for some time [5]. Multiple Dengue vaccines are in the pipeline, some licensed for use but do not provide consistent evidence of benefit across all age groups nor serotype cross-immunity [6].

## Discussion

Since there are no effective therapeutic interventions for Dengue, many alternative remedies (natural and herbal) have been utilized as adjuvant. Carica Papaya Leaf Extract (CPLE) is used off-label, which means unapproved clinical uses in treating Dengue-like symptoms (on a higher note over socializing platforms), which took place very recently. Although papaya leaf crude extract is still widely used, commercial formulations containing papaya leaf extract have also been marketed in some regions. It is a common plant in the tropics worldwide and includes edible fruits known as papaya and Carica papaya (CP). While it is rooted in Mexico and the South Americas, its growth has been exponential after being naturalized to nations from the south of Asia to West Africa. The leaves of the plant have high amounts and concentrations of biologically active compounds such as papain, caricain, chymopapain, and glycine endopeptidase. CPLE has antiviral actions and thus might correct hematological defects that can represent the pathological basis for its use in the treatment of Dengue-like activity as an antioxidant and free scavenger and hemocompatibility property including improvement in red cell membrane stabilization [7]. Additionally, CP leaf extract-derived flavonoids have been shown to act as a protease inhibitor vital for the complete formation of virus particles [8].

A randomized trial of 39 patients with DF or Dengue Hemorrhagic Fever (DHF) in Pakistan investigated the effect on platelet counts. In another study, the platelet count in DF was not found to be influenced by papaya leaf extract [9]. This was a multicenter, randomized, open-label controlled experiment of 80 Dengue patients in Indonesia, a study testing the effectiveness of CPLE capsules amongst DHF victims. The primary outcomes reported were a rise in platelets, a fall in hematocrit, and discharge/period [10]. Mean Platelet Count Difference in Dengue Patients Treated with CP Leaf Extract Juice After 3 Days; A Randomized Controlled Study (Open Label) of Three Different doses conducted on a Population of Malaysia, CPLE was found to induce a significant platelet count increase only in cases of DF and DHF [11]. Treatment of Dengue infection (DF and DHF grade I) with CP leaf extract, described in five more studies from India on mean platelet counts, showed an increment of platelet level in the interventional group [12–16].

## Conclusion

In conclusion, There has been a growing number of increasingly older cases, and much more is yet to be learned about Dengue in the elderly. The diagnostic criteria for this patient population need to be refined to facilitate an earlier diagnosis, predict disease severity, and identify patients likely to progress into the more severe stage. This population would likely require altered management strategies because of the increased co-morbidities. The place for Dengue vaccines in this category is unclear, and more safety and efficacy studies are required. While a few have found possible benefits to CP, there is not enough evidence to suggest that CP extracts should be used universally for Dengue management. Currently, a limited amount of high-to-moderate quality clinical evidence could be used to support the use of CPLE in treating human Dengue illness. Although platelet counts are increased and hospital stays may be reduced, it is difficult to support the prescription of specific antioxidants because so much of the research on this topic had methodological flaws. Insufficient scientific evidence supports using CP extract to treat Dengue fever in routine practice. Therefore, a randomized controlled trial (RCT) should be planned and powered to evaluate the impact of CP on the following factors: preventing plasma leakage (for which platelet count variations may serve as a surrogate marker), preventing shock and multi-organ dysfunction, necessitating blood and blood product transfusions, necessitating Intensive Care Unit (ICU) admission, and length of ICU and hospital stay. It is also crucial to assess the benefit of CPLE in increasing platelet counts, focusing on correlating these changes with the timing of clinical illness. This approach will help determine if CPLE can effectively manage thrombocytopenia in dengue patients at various stages of the disease. Thus, the potential of Carica papaya as a cost-effective intervention in the dengue-endemic areas is significant, and further research could unlock new avenues for its application in public health.

## References

[1] Rajapakse S, de Silva NL, Weeratunga P, et al. Carica papaya extract in dengue: a systematic review and meta-analysis. BMC Complement Altern Med. 2019;19(1). doi: 10.1186/s12906-019-2678-2 [PMC Free Article] [PubMed] [Google Scholar]. PMC6788024 31601215

[2] Macias AE, Werneck GL, Castro R, et al. Mortality among hospitalized dengue patients with comorbidities in Mexico, Brazil, and Colombia. Am J Trop Med Hyg. 2021 May 10;105(1):102–4. doi: 10.4269/ajtmh.20-1163 PMID: 33970884; PMCID: PMC8274750. 33970884 PMC8274750

[3] Rajapakse S, Rodrigo C, Maduranga S, et al. Corticosteroids in the treatment of dengue shock syndrome. Infect Drug Resist. Published online 2014 May:137. doi: 10.2147/IDR.S55380 [PMC Free Article] [PubMed] [Google Scholar]. 24899817 PMC4038529

[4] Rajapakse S. Intravenous immunoglobulins in the treatment of dengue illness. Trans R Soc Trop Med Hyg. 2009;103(9):867–870. doi: 10.1016/j.trstmh.2008.12.011 [PubMed] [Google Scholar]. 19203771

[5] Lee TH, Lee LK, Lye DC, et al. Current management of severe dengue infection. Expert Rev Anti Infect Ther. 2017;15(1):67–78. doi: 10.1080/14787210.2017.1248405 [PubMed] [Google Scholar]. 27786589

[6] Hadinegoro SR, Arredondo-García JL, Capeding MR, et al. Efficacy and long-term safety of a dengue vaccine in regions of endemic disease. N Engl J Med. 2015;373(13):1195–1206. doi: 10.1056/NEJMoa1506223 [PubMed] [Google Scholar]. 26214039

[7] Ranasinghe P, Ranasinghe P, Sirimal Premakumara G, et al. In vitro erythrocyte membrane stabilization properties of Carica papaya L. leaf extracts. Phcog Res. 2012;4(4):196. doi: 10.4103/0974-8490.102261 [PMC Free Article] [PubMed] [Google Scholar]. 23225962 PMC3510871

[8] Senthilvel P, Lavanya P, Kumar KM, et al. Flavonoid from Carica papaya inhibits NS2B-NS3 protease and prevents dengue 2 viral assembly. Bioinformation. 2013;9(18):889–895. doi: 10.6026/97320630009889 [PMC Free Article] [PubMed] [Google Scholar]. 24307765 PMC3842573

[9] Charan J, Saxena D, Goyal J, et al. Efficacy and safety of Carica papaya leaf extract in the dengue: a systematic review and meta-analysis. Int J App Basic Med Res. 2016;6(4):249. doi: 10.4103/2229-516X.192596 [PMC Free Article] [PubMed] [Google Scholar]. PMC5108100 27857891

[10] Yunita F, Hanani E, Kristianto J. The effect of carica papaya L. Leaves extract capsules on platelets count and hematocrit level in dengue fever patient. 2012. [Google Scholar].

[11] Subenthiran S, Choon TC, Cheong KC, et al. Carica papaya leaves juice significantly accelerates the rate of increase in platelet count among patients with dengue fever and dengue Haemorrhagic fever. Evidence-Based Complementary Alternative Med. 2013;2013:1–7. doi: 10.1155/2013/616737 [PMC Free Article] [PubMed] [Google Scholar]. PMC3638585 23662145

[12] Abhishek G, Vinod T, Malik SK, et al. Efficacy of carica papaya leaf extract in treating thrombocytopenia in cases of dengue. J Adv Res Med Sci. 2015;(7):01–04.

[13] Gowda A, Kumar NV, Kasture P, et al. A pilot study to evaluate the effectiveness of carica papaya leaf extract in increasing the platelet count in cases of dengue with thrombocytopenia. Published 2015. [Google Scholar].

[14] Gadhwal AK, Ankit BS, Chahar C, et al. Effect of Carica papaya leaf extract capsule on platelet count in patients of dengue fever with thrombocytopenia. J Assoc Physicians India. 2016;64(6):22–26. [PubMed] [Google Scholar]. 27739263

[15] Kasture PN, Nagabhushan KH, Kumar A. A multi-centric, double-blind, placebo-controlled, randomized, prospective study to evaluate the efficacy and safety of Carica papaya leaf extract, as empirical therapy for thrombocytopenia associated with Dengue Fever. J Assoc Physicians India. 2016;64(6):15–20. [PubMed] [Google Scholar]. 27739262

[16] Doddamane K, Kumar D. Role of carica papaya leaf product in improving the platelet count role of carica papaya leaf product in improving the platelet count in patients with dengue fever. Int J Res Med. 2017;6(2). [Google Scholar].

